# Cutaneous leishmaniasis in sub-Saharan Africa: a systematic review of *Leishmania* species, vectors and reservoirs

**DOI:** 10.1186/s13071-024-06381-8

**Published:** 2024-07-24

**Authors:** Romain Blaizot, Gregoire Pasquier, Abdoulaye Kassoum Kone, Alexandre Duvignaud, Magalie Demar

**Affiliations:** 1 Department of Dermatology, Centre Hospitalier de Cayenne, Cayenne, French Guiana; 2 National Reference Center for Leishmaniasis–Centre Hospitalier de Cayenne, Cayenne, French Guiana; 3 Centre Hospitalier Universitaire de Montpellier, Montpellier, France; 4 UMR 1019 TBIP–Tropical Biomes and Immunopathophysiology, Université de Guyane, Cayenne, French Guiana; 5 Malaria Research and Training Center, University of Sciences, Techniques, and Technologies, Bamako, Mali; 6 Department of Infectious Diseases and Tropical Medicine, Hôpital Pellegrin, Centre Hospitalier Universitaire de Bordeaux, Bordeaux, France; 7 Inserm UMR 1219, IRD EMR 271, Bordeaux Population Health, Université de Bordeaux, Bordeaux, France; 8 Laboratory of Parasitology-Mycology, Centre Hospitalier de Cayenne, Cayenne, French Guiana

**Keywords:** Cutaneous leishmaniasis, Sub-Saharan Africa, Review, One Health, Vectors, Reservoirs

## Abstract

**Background:**

Cutaneous leishmaniasis (CL) is understudied in sub-Saharan Africa. The epidemiology of CL is determined by the species involved in its transmission. Our objectives were to systematically review available data on the species of *Leishmania*, along with vectors and reservoirs involved in the occurrence of human cases of CL in sub-Saharan Africa, and to discuss implications for case management and future research.

**Methods:**

We systematically searched PubMed, Scopus, Cochrane and African Index Medicus. There was no restriction on language or date of publication. The review was conducted according to PRISMA guidelines and was registered on PROSPERO (CRD42022384157).

**Results:**

In total, 188 published studies and 37 reports from the grey literature were included. An upward trend was observed, with 45.7% of studies published after 2010. East Africa (55.1%) represented a much greater number of publications than West Africa (33.3%). In East Africa, the identification of reservoirs for *Leishmania tropica* remains unclear. This species also represents a therapeutic challenge, as it is often resistant to meglumine antimoniate. In Sudan, the presence of hybrids between *Leishmania donovani* and strictly cutaneous species could lead to important epidemiological changes. In Ghana, the emergence of CL in the recent past could involve rare species belonging to the *Leishmania* subgenus *Mundinia*. The area of transmission of *Leishmania major* could expand beyond the Sahelian zone, with scattered reports in forested areas. While the *L. major*–*Phlebotomus duboscqi*–rodent complex may not be the only cycle in the dry areas of West Africa, the role of dogs as a potential reservoir for *Leishmania* species with cutaneous tropism in this subregion should be clarified. Meglumine antimoniate was the most frequently reported treatment, but physical methods and systemic agents such as ketoconazole and metronidazole were also used empirically to treat *L. major* infections.

**Conclusions:**

Though the number of studies on the topic has increased recently, there is an important need for intersectional research to further decipher the *Leishmania* species involved in human cases of CL as well as the corresponding vectors and reservoirs, and environmental factors that impact transmission dynamics. The development of molecular biology in sub-Saharan Africa could help in leveraging diagnostic and research capacities and improving the management of human cases through personalized treatment strategies.

**Graphical Abstract:**

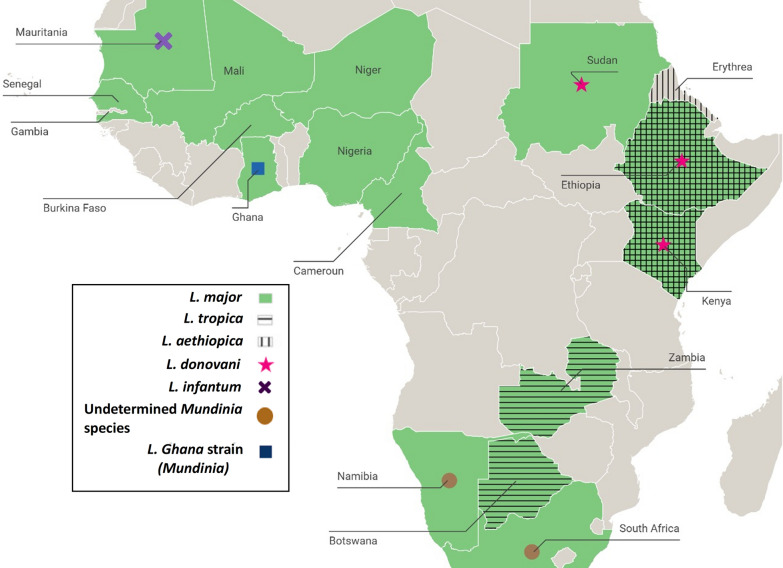

**Supplementary Information:**

The online version contains supplementary material available at 10.1186/s13071-024-06381-8.

## Background

Cutaneous leishmaniasis (CL) is a neglected tropical disease transmitted to humans through the bite of sand flies previously infected on mammalian reservoirs [1]. Worldwide, more than 200,000 new cases are reported annually [1]. Areas of CL endemicity can overlap those of visceral leishmaniasis (VL), a systemic form of *Leishmania* infection caused by specific species (*Leishmania infantum* and *Leishmania donovani*). Post-Kala-Azar dermal leishmaniasis (PKDL) is a post-treatment complication of VL, due to increased inflammation around parasites surviving in the skin [2]. The pathophysiology and epidemiology of PKDL is therefore very different from that of CL.

CL is extensively described in the Americas, in the Near and Middle East and in North Africa. Sub-Saharan Africa (SSA) is also affected, as 26 different countries have reported cases at some point [3]. However, there is a large research gap concerning the epidemiology of the disease in this region of the world, where CL is probably under-diagnosed and under-declared. A recent review of human studies on CL in SSA highlighted the lack of data on the exact incidence of CL and factors associated to its transmission [3]. There is also a large research gap in the knowledge of the different *Leishmania* species and strains involved, as well as the associated hosts and vectors.

The clinical presentation of CL in humans may vary on the basis of several factors, including the host’s immune response and characteristics of the parasitic species and strain involved. For example, in the Americas, *Leishmania braziliensis* is the species most likely to cause mucosal lesions [4]. *Leishmania amazonensis* is typically associated with diffuse leishmaniasis, a very peculiar form of the disease presenting with numerous anergic nodules and high parasitic loads [5]. Within the same species, specific strains can also be associated with clinical features such as dissemination, number of lesions or resistance to antileishmanial drugs, as demonstrated in *L. braziliensis* and *Leishmania guyanensis* [6, 7]. Therefore, mapping the distribution of human-infecting *Leishmania* species across SSA can have a significant impact on the understanding of the disease and clinical case management.

In addition, the epidemiology of CL depends on the distribution of different vectors and reservoirs. Knowing which sand flies and which mammals are involved in the transmission cycle of a particular *Leishmania* species in SSA could aid in understanding the spatial and temporal evolution in incident human cases. Due to a lack of laboratory facilities and funding, entomological, biomolecular and veterinary studies are scarce in SSA. Recent data may challenge old assumptions on parasitic cycles which have often benefited from few studies since the era of colonial medicine. For example, recent outbreaks of CL have been reported in Ghana [8]. This country does not belong to the traditional “CL belt” of semi-Sahelian areas which were deemed the only endemic regions in West Africa. It is possible that specific forms of CL caused by rare species might exist in unsuspected ecosystems such as the forest hills of eastern Ghana. Finally, climate change and other modifications in African ecosystems could shift the epidemiology of CL and cause the occurrence of new CL foci. Thus, identifying the different species involved at each step of the parasitic cycle through a One Health approach is necessary to optimize public health interventions targeting CL in SSA.

Our aim was to perform a systematic review of the literature regarding *Leishmania* species, vectors and reservoirs in SSA, and to discuss the implications of available data for clinical case management and future research.

## Methods

We searched the following databases: Cochrane register, including Skin and Infectious Diseases groups; PubMed/NCBI; MEDLINE/Ovid; African Index Medicus; and Scopus. Subsequent searches were made using a combination of keywords ((“sergentomyia” OR “phlebotom*” OR “sandfly*”) OR (“leishmania major “ OR “leishmania tropica” OR “leishmania chancei” OR “leishmania infantum” OR “leishmania enriettii “ OR “leishmania aethiopica “ OR “leishmania donovani “)) AND (“cutaneous leish*”) AND (“Africa, South of Sahara” OR each individual sub-Saharan country according to the World Bank classification).

Concerning grey literature, the following sources were used: conference proceedings (*World Leish*; *Annual Meeting of the American Society of Tropical Medicine and Hygiene*; *Journées Dermatologiques de Paris*; *Journées Nationales d’Infectiologie*; *European Congress of Clinical Microbiology & Infectious Diseases*) and reports/fact sheets from the World Health Organization.

There was no restriction on language, and the search was limited to data published before 1 January 2023. Studies were included when reporting data on vectors, hosts or *Leishmania* species in the appropriate geographical area (SSA: meaning all of Africa except North Africa). All study types were included, including reviews, original articles, research letters, case series and case reports, and conference proceedings. Articles on animals, vectors and humans were eligible. Studies describing data on human CL without any information on the species involved or with information on One Health aspects only were excluded from the formal analysis but could be used for the discussion. Studies which did not indicate the country of origin of samples/patients and studies concerning only VL or PKDL were excluded, as well as studies with no implication for human health.

Two investigators (RB and AD) independently screened the titles and abstracts extracted from the different databases. Records were excluded when titles/abstracts indicated no link to CL, SSA or human health. Full texts were then sought for retrieval and assessed for eligibility. Reports were not assessed for eligibility when full texts were not available. Discordances were resolved through discussion with a third investigator (MD). Relevant data from the grey literature, such as congress proceedings, were retained even without any corresponding published full-text article.

Data concerning *Leishmania* species and identified vectors and reservoirs were presented by subregion and by country. Concerning data on possible vectors, information regarding the incrimination criteria was given [9].

This study was conducted according to the Preferred Reporting Items for Systematic reviews and Meta-Analyses (PRISMA) guidelines [10] and registered on PROSPERO with accession number CRD42022384157.

## Results

**Fig. 1:**
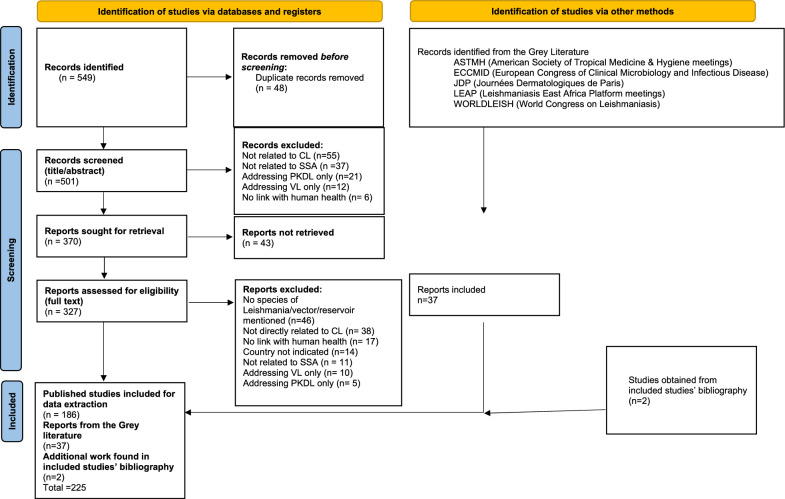
PRISMA 2020 flow diagram, systematic review of species of *Leishmania*, vectors and reservoirs involved in human cutaneous leishmaniasis in sub-Saharan Africa, 2023

In total, 188 studies were included (186 from the queries and two that were identified in the bibliography of included studies), along with 37 reports from the grey literature. A PRISMA flowchart illustrating the process of selection and inclusion is presented in Fig. 1.

The oldest study was published in 1956, the most recent in 2023 (published online in late 2022). An upward trend in publication was observed, with 35 studies (18.7%) published between 1956 and 1990, 67 (35.6%) between 1990 and 2010, and 86 (45.7%) after 2010. Concerning the topic of studies, 57 articles and two grey literature reports were focused on human results, often consisting of case reports, case series or clinical and epidemiological reports; 43 articles and 15 reports were entomological; 32 articles and seven reports were focused on *Leishmania* species or strains, with description of molecular biology or zymodeme analysis; 14 articles and five reports were focused on reservoirs; four articles and one report described data on ecological changes that might influence the epidemiology of CL through its vectors or reservoirs; and three articles provided histopathological data corresponding to the clinical presentations of infections by different *Leishmania* species. Lastly, 19 articles and four reports described new CL foci or outbreaks with clinical and One Health aspects, while 16 studies and three reports were general reviews.

The number of studies included by country is presented in Table 1. East Africa was by far the most frequently studied region (124 studies and reports, 55.1%), with Senegal, Burkina Faso and Mali being the countries with the most available data within West Africa (75 studies and reports, 33.3%). Cameroon was the only studied country in Central Africa. Of note, numerous congress proceedings reporting data from Ghana, a country that has long been considered non-endemic, were published recently.

**Table 1:** Number of published studies and grey literature reports included by country

Country	Number of published studies included	Number of grey literature reports included	Total
East Africa	112	13	124
Ethiopia	47	2	48
Kenya	35	9	44
Sudan	25	2	27
Eritrea	1		1
Reviews/case series on East Africa	4		4
West Africa	58	19	75
Senegal	22		22
Burkina Faso	9	1	10
Mali	9	4	13
Ghana	10	13	21
Gambia	3		3
Mauritania	1		1
Niger	1		1
Nigeria	1	1	2
Reviews/case series on West Africa	2		2
Central Africa	9	2	11
Cameroon	9	2	11
Southern Africa	6	8	8
Namibia	2	2	4
Republic of South Africa	2		2
Botswana	1		1
Zambia	1		1
General reviews and series	3	1	4
Total			225

Fact sheets summarizing all available data for each country are available in Supplementary Materials 1–14. Detailed data are presented below for each country regarding clinical presentations, the different isolated species of *Leishmania*, vectors and reservoirs, and ecological implications in terms of possible epidemiological shifts.

**Fig. 2:**
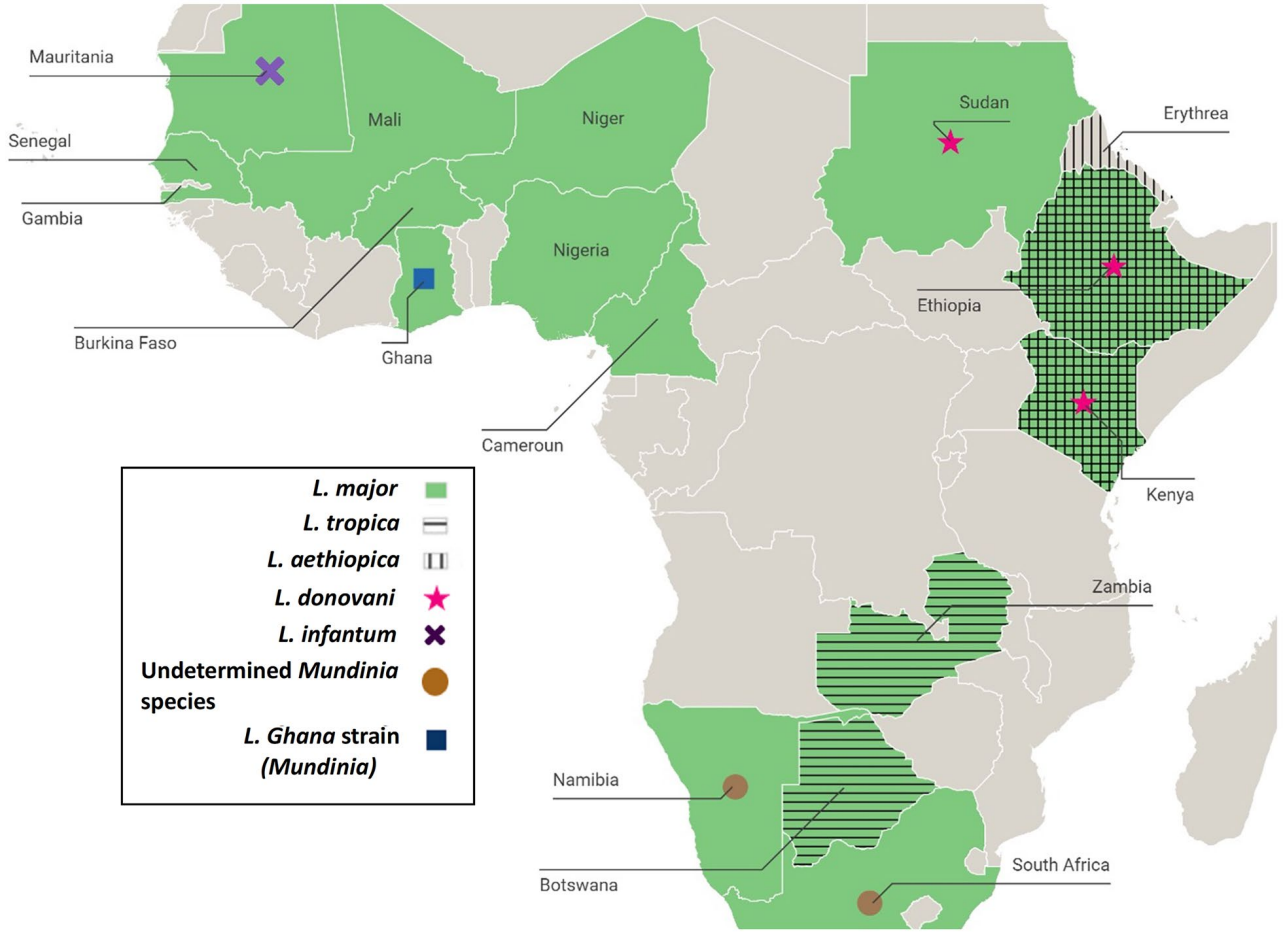
Map of the different sub-Saharan African countries with corresponding cases of *Leishmania* species reported in humans, 2023

A map gathering all identified *Leishmania* species in the different countries of SSA is presented in Fig. 2.

**Fig. 3:**
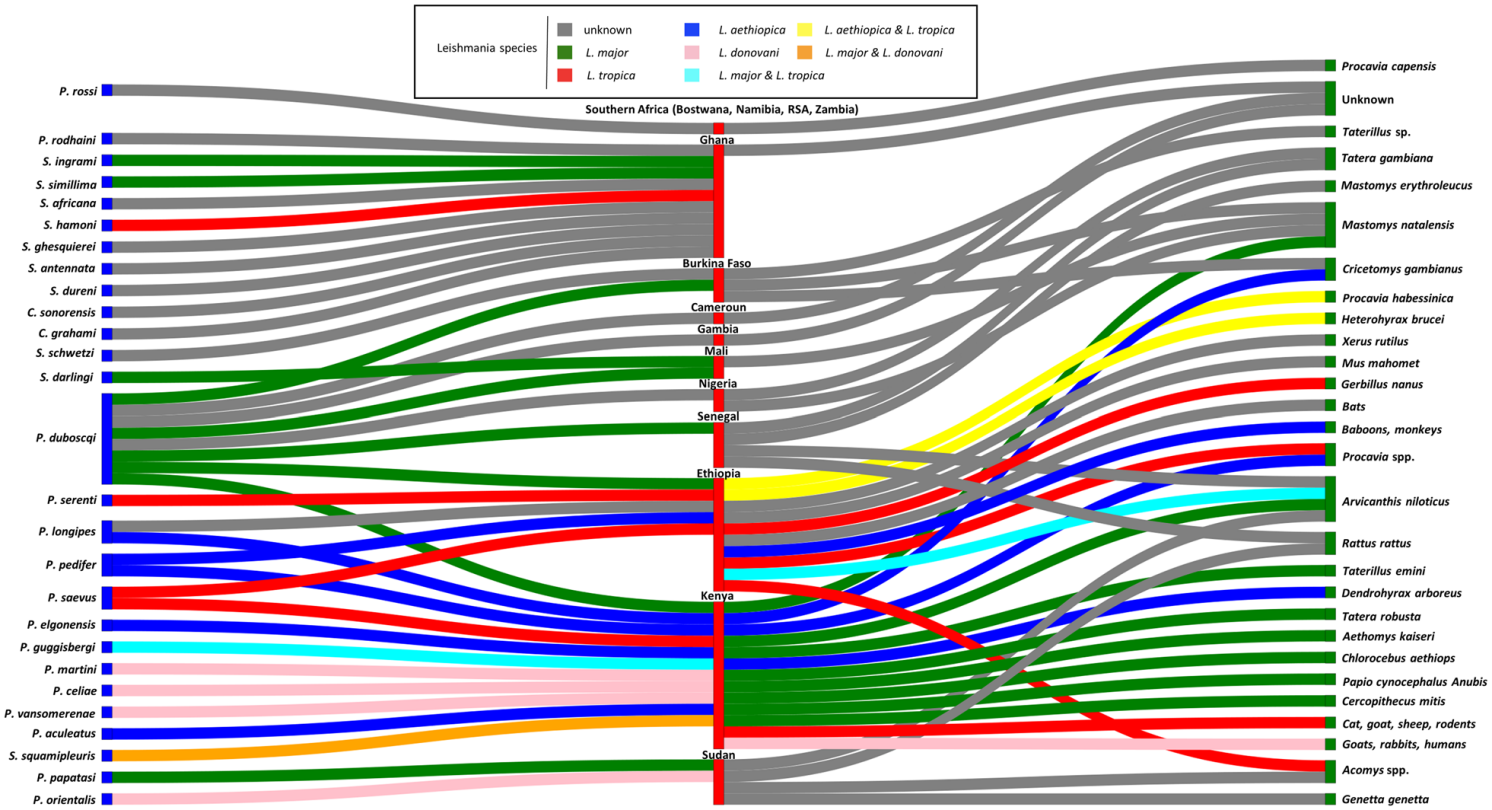
Sankey diagram showing the reported links for each species of *Leishmania*, and vector and reservoir involved in cutaneous leishmaniasis in sub-Saharan Africa; vectors are on the left of the diagram, reservoirs on the right; countries of isolation are in the center of the diagram; *Leishmania* species are indicated by different colours

A Sankey diagram is also available as Fig. 3, presenting the different species of vectors and reservoirs, in relation to the corresponding *Leishmania* species and countries involved.

### East Africa

#### Ethiopia

Ethiopia is one of the countries with the largest number of available studies and, similar to the rest of East Africa, is characterized by the presence of numerous species of *Leishmania*, vectors and reservoirs. The corresponding clinical presentation more often includes patches, plaques and nodules rather than ulcers, and can be localized, mucocutaneous [11, 12] or diffuse [13].

The parasite causing CL in Ethiopia was thought to be *Leishmania tropica* until Bray et al. identified *Leishmania aethiopica* as the main species in 1973. *Leishmania tropica*, *Leishmania major*, and *L. donovani* are also responsible for human cases of CL in the country [14, 15]. The epidemiology of CL in Ethiopia is well differentiated, with *L. aethiopica* being responsible for CL in the Ethiopian Highlands, while *L. major* and *L. tropica* cause CL in the lowlands areas [16].

The vectors of *L. aethiopica* are *Phlebotomus longipes* in central and northern Ethiopia and *Phlebotomus pedifer in* the south-western part of the country [17]. In the lowlands, *L. tropica* has been detected in *Phlebotomus serenti* and *Phlebotomus saevus* [18], while *L. major* was isolated in *Phlebotomus duboscqi* [19].

Identified reservoirs for *L. aethiopica* are the rock hyraxes *Procavia habessinica* and *Heterohyrax brucei* [20]. Baboons and other monkeys can be infected in the laboratory with *L. aethiopica* but are not common enough around human dwellings to represent a significant reservoir for transmission to humans [21, 22]. *Leishmania tropica* was detected in wild rodents (*Acomys*, *Arvicanthis*, *Gerbillus*) in 2014 in the VL and CL endemic area of Awash-Metehara (near Addis-Ababa, east-central Ethiopia) and the VL endemic areas around Konso and Yabelo (South Ethiopia) [23]. This species was also identified in *Arvicanthis* sp., *Gerbillus nanus* and *Acomys* spp. [24] in different areas of Ethiopia. Rock hyrax and bush hyrax are also thought to be reservoirs for this species [24]. Of note, *L. tropica* and *L. major* have been detected in small samples of the bats *Cardioderma cor* and *Nycteris hispida*, respectively. These bats nest in caves, which are ideal for sand fly breeding and could therefore represent other significant hosts [25]. *Leishmania major* has also been detected in *Arvicanthis niloticus* [24, 26].

Small new zoonotic foci of CL have been reported in the south-eastern part of the capital Addis-Ababa. Their occurrence was attributed to the uncontrolled urbanization of previously rural areas, and the subsequent disruption of hyrax habitat. The establishment of new contacts between humans and hyraxes could have led to the emergence of CL. The authors suggested the possibility that even if hyraxes were to disappear due to anthropization, the disease could self-sustain with humans as a new parasite reservoir [22]. However, most studies on leishmaniasis in Ethiopia considered that humans were not ideally suited for *L. aethiopica* and *L. tropica* and became infected accidently [21].

Human cases of CL mostly occur along caves or in rocky spots in rural areas. The development of *P. pedifer* and of the parasite in the vector requires sharp variations in rainfall as well as moderate temperature. For this reason, the Ethiopian Lowlands are unsuitable due to high temperatures and small variations in rainfall, while higher altitudes are too cold. Due to global warming, rainfall is predicted to become erratic, droughts and floods could become more frequent, and the mean temperature could increase. This could allow CL to develop in previously non-endemic areas where the hyrax reservoirs are present and where the population is still non-immune [17].

#### Eritrea

Strikingly, there are very few data on CL in the former Ethiopian province of Eritrea. Only one case has been reported to date, in a German immunocompromised traveller returning from Eritrea. He was found infected with *L. aethiopica* and was successfully treated with liposomal amphotericin B [27]. One could imagine that the local political context makes it difficult to conduct research studies and could hypothesize that the epidemiology of CL in the country is similar to neighbouring areas of Ethiopia.

#### Kenya

CL in Kenya is associated with typical oriental sores, sometimes multiple papules [28]. A case of CL caused by *L. donovani* was also reported in 1993, in a young man from the Nyandarua District who presented with a single lesion on the nose and required several cures of sodium stibogluconate [29].

The species responsible for CL in Kenya include *L. aethiopica* in the Mount Elgon area, *L. major* in the Baringo District of the Rift Valley and *L. tropica* in the central highlands [28, 30–32].

Concerning *L. aethiopica*, implicated vectors include *P. longipes* and *P. pedifer*, while scarce data also suggest that *Phlebotomus elgonensis* [31] and *Phlebotomus celiae* may be involved in transmission [33]. Notably, in 1970, parasites were extracted from *P. longipes* samples captured in Mount Elgon and administered to hamsters, leading to the development of cutaneous nodules. *Phlebotomus longipes* were also fed on human lesions and developed important parasitic charges [34]. In the Mount Elgon region, *P. pedifer* and *P. longipes* feed on the rock hyrax *Procavia johnstoni*, on tree hyrax *Dendrohyrax arboreus*, and on the giant rat *Cricetomys gambianus*, which are the known reservoirs for *L. aethiopica* in this country [35].

Concerning *L. tropica*, it has been isolated from *Phlebotomus guggisbergi* (a cave-dwelling sand fly) and *Phlebotomus aculeatus* (in caves and rock crevices) [35], and more recently from *P. saevus* [36]. The development of *L. tropica* in *P. guggisbergi* has also been demonstrated [37]. The ability of *P. duboscqi* from Kenya to carry *L. tropica* has been experimentally demonstrated [38], though infected specimen have yet to be found in the field. Although rock hyraxes are reservoirs of *L. aethiopica*, their role in the transmission of *L. tropica* remains unclear. While this species is anthroponotic in North Africa, the high infection rate in *P. guggisbergi* suggests the existence of an animal reservoir, as this species of phlebotomine has been shown to feed more often on larger mammals (cats, goats and sheep) than on rodents in Kenya [39]. However, populations of *P. guggisbergi* infected by *L. aethiopica* are mostly found around caves and natural habitats, and are rarely in contact with cattle. Therefore, another less anthropized reservoir of *L. tropica* should be looked for in Kenya [40]. It has been suggested that the massive elimination of rodents could lead *P. duboscqi* to change their feeding behaviour and bite on humans instead [41].

Concerning *L. major*, it was long thought that *P. duboscqi*, which lives in animal burrows, was the only vector [35, 42]. However, a recent study in Nakuru County showed for the first time the presence of *L. major* in *P. guggisbergi*. Blood meal analysis suggested contact with humans and rodents [36, 43]. In the Merti area, which was deemed endemic for the sole VL [36], specimens of *Sergentomyia squamipleuris* have been found positive for *L. major* and *L. donovani*, with proven blood meals on humans and rodents.

As in other countries of SSA, rodents seem to be the reservoir for *L. major* in Kenya. In the Baringo district, *L. major* has been identified by isoenzyme analysis in samples of *Tatera robusta*, *A. niloticus* and *Mastomys natalensis* [44]. But *L. major* antibodies have also been found in primates such as *Chlorocebus aethiops* (vervet monkeys), *Papio cynocephalus anubis* (olive baboons) and *Cercopithecus mitis* (Sykes’ monkeys), captured in five different endemic regions of Kenya [45]. *Leishmania major* was also isolated in culture from a skin papule of a Kenyan vervet monkey in 1987 [46], though the specificity of the serological assay used (antibody enzyme-linked immunosorbent assay [Ab-ELISA], western blot and recall lymphocyte proliferation assay) and the possibility of cross-reactions with other species should be discussed.

#### Sudan

Three clinical types of CL have been reported in Sudan: the ulcerative type, the nodulo-ulcerative/nodular form and the non-ulcerated diffuse form.

In this country, *L. major* and *L. donovani* have been historically reported as responsible for both CL and mucosal leishmaniasis (ML). Concerning CL, El Hassan et al. demonstrated that some skin lesions with positive polymerase chain reaction (PCR) for *L. donovani* were mistakenly reported as genuine oriental sore by clinicians but were actually inoculation sores preceding VL [47]. Interestingly, in 2014, Babiker et al. described cases of concomitant infections by *L. major* and *L. donovani* in some genuine CL lesions [48]. PCR of the internal transcribed spacer (ITS) gene followed by sequencing and phylogeny identified sequences from the two species. The authors suggested two hypotheses, either the co-existence of the two species in *Phlebotomus papatasi* and concomitant transmission to humans, or the emergence of hybrids of both species. Those hybrids could give birth to strictly cutaneous presentations of *L. donovani*, without visceral involvement [48]. Regarding the non-ulcerated diffuse phenotype, El Hassan et al. also showed that some misdiagnosed cases of PKDL actually turned out to be CL with positive *L. major* PCR [49]. Concerning the clinical presentations, several cases reported as “diffuse CL” (DCL) were actually disseminated but without the corresponding histopathological pattern of dysfunctional immune anergic response that characterizes DCL [49]. Lastly, it seems that Sudanese ML is not preceded by cutaneous lesions, as happens in South America, but rather by VL caused by *L. donovani* [48, 50]. Some authors have suggested that Sudanese ML and VL could be two opposite extremes in the spectrum of a common clinical entity. On the other hand, *L. major* has been identified in mucosal lesions in Sudan but never as an agent of VL. In addition, *L. major* was isolated in one patient without cutaneous lesions and with no previous history of VL, suggesting that this species could also be responsible for strictly mucosal disease in Sudan [51]. A much wider spectrum of rarer clinical presentations was also reported, such as sporotrichoid, cheilitis, and mycetoma-like lesions [47, 52]. *Leishmania major* zymodeme LON-1 is the most frequently reported strain in Sudan, notably in the 1986 Khartoum outbreak, though MON-74 has also been reported [53].

Since 1955, *P. papatasi* has been suspected as a vector of CL in Sudan, as it is highly prevalent in endemic areas such as in Darfur or in North Khartoum, and was captured close to outbreak locations. Specimens were found infected with *L. major* in 1997, and a blood meal analysis suggested that humans were the main prey, though this sand fly also fed on rodents [47]. Small populations of *Phlebotomus sergenti* and *P. duboscqi* (known to be a vector of *L. major* in West Africa) have also been detected in Sudan, but their role in local transmission remains unclear [47].

In 1966, Adler et al. found that the serological profile of three strains isolated from human CL cases was identical to those obtained from two patients with VL, from infected *Phlebotomus orientalis* and from specimens of *Rattus rattus*, *Acomys* sp. and *A. niloticus* [54]. These results suggested that similar strains were involved in both CL and VL in Sudan. However, the authors used a technique of culture followed by haemagglutination tests that did not allow them to properly identify this species.

In 1990, *Leishmania* spp. were identified in 13 *A. niloticus* and one *Genetta genetta*. One isolate from *A. niloticus* was identified as *L. major* zymodeme LON-1 [47].

### West Africa

#### Senegal

Although foci are localized, ulcero-crusted CL is the most frequent form in Senegal. Several cases of pseudo-lepromatous DCL or pseudo-tumoral vegetant CL have also been reported in the country [55–57]. In one case, *L. major* was identified [55]. A few cases of infection by *L. infantum* have been reported combining a VL phenotype with either lupoid or ulcerous and crusty cutaneous lesions [56], or associated with strictly cutaneous lesions. The latter occurred in a human immunodeficiency virus (HIV)-infected child [58]. However, these clinical patterns of *L. infantum* seem very rare.

Due to ancient and numerous studies, Senegal exemplifies the parasitic cycle of *L. major* in West Africa, where this species is transmitted from rodents to humans by *P. duboscqi*, in dry, rural areas. CL was first reported in Senegal in 1933. In the 1970s, similar strains of *L. major* were identified in rodents and specimens of *P. duboscqi* collected in the same burrows [59]. A recent entomological study conducted in the Thiès region showed a geographical correlation between the seroprevalence of canine leishmaniasis and the proportion of *Sergentomyia dubia*, a suspected vector of *L. infantum* canine leishmaniasis in this area [60]. Concerning reservoirs, *L. major* was isolated in captured *Tatera gambiana* and *Mastomys erythroleucus*. *Arvicanthis niloticus* is considered an accidental reservoir due to lower infection rates [61, 62]. In addition, *A. niloticus* dwells in nets rather than in the burrows where the vector is found [59]. Recently, strains of *L. major* identical to those circulating in humans have been detected in the invasive species *R. rattus*. Although its role in the transmission to humans has yet to be determined, these data suggest that the black rat could lead to new CL outbreaks in more urban areas than the current rural foci where wild endemic rodents act as the main reservoirs [63].

#### The Gambia

CL is rarely reported in the Gambia, with only a few published cases [64, 65]. No *Leishmania* strain has yet been isolated from humans. Entomological and reservoir studies have shown the presence in areas where human clinical cases were reported of sand flies such as *P. duboscqi* and rodents similar to those involved in neighbouring Senegal, but without detection of *Leishmania* [64].

#### Burkina Faso

Although the form of CL usually reported in the country is ulcero-crusted [66], several cases of *L. major* diffuse pseudo-lepromatous CL, with lymph nodes or bone marrow involvement, have been reported in HIV-infected patients [67, 68].

*Leishmania major* is, to date, the only species detected in Burkina Faso, both during the large outbreak in Ouagadougou [66] and in the west of the country [66, 69, 70]. As in Mali, both zymodemes MON-74 (reported only in Mali and Burkina Faso) and the more frequent MON-76 (found in Senegal, West Africa and the Near East) have been reported [67, 69]. *Phlebotomus duboscqi* is the only sand fly of the *Phlebotomus* genus captured in the Ouagadougou focus, but there was no specimen positive for *L. major* [71]. Interestingly, samples of *Sergentomyia* (notably *Sergentomyia schwetzi*, known for its anthropophilic behaviour) have also been captured in such foci of CL, but their role in transmission to humans remains unclear.

Identified reservoirs of *L. major* in Burkina include rodents, with positive PCR in *M. natalensis*, *Taterillus* sp. and *C. gambianus* in different locations of the Ouagadougou area [72]. More recently, *L. major* has also been detected serologically and by PCR in dogs captured in Bobo-Dioulasso [73].

#### Mali

*Leishmania major* is the only species detected in Mali [74, 75], with both MON-74 and MON-76 zymodemes [69]. It has been isolated in *P. duboscqi* [75] and, more recently, *Sergentomyia darlingi* [76]. Human blood was identified in both these vector species, confirming their anthropophilic behaviour [76].

#### Mauritania

The ulcero-crusted form is the most frequent. *Leishmania major* is the most common species in Mauritania [77, 78], while *L. infantum* was also identified in 24% of cases in a recent study [77] of patients infected in the south of the country. There are no data on vectors. Concerning reservoirs, a study conducted in 2019 in the Malian district of Barouéli, along the Mauritanian border, reported the presence of anti-*Leishmania* antibodies in *M. natalensis*, but no parasite could be retrieved from the rodents. Blood meal analysis showed that *P. duboscqi* fed mostly on chickens, goats and humans [79].

#### Niger

Two main clinical forms have been reported, the dry ulcero-crusted one and the humid-ulcerous one [80], as well as sporotrichoid cases [81]. *Leishmania major* (zymodeme MON-1) is the only species isolated in Niger [80]. There are no data on vectors or reservoirs.

#### Nigeria

The presence of CL in the northern semi-Sahelian regions of Nigeria has been confirmed by microscopy [82]. To date, we have retrieved only one congress report mentioning the identification of *L. major* by PCR in a skin ulcer [83]. Entomological studies have identified many sand flies of the *Phlebotomus* and *Sergentomyia* genera, without any positive *Leishmania* PCR [82]. Positive microscopy has been reported in livers and spleens of *M. natalensis* and *T. gambiana* [84].

#### Ghana

Lesions of CL in Ghana have been described as usually circular, crusted or ulcerated, located on uncovered body areas. Initial reports described many multiple lesions [8], while more recent studies reported no case of diffuse or disseminated presentation [85]. Though all age classes were described in the first studies, children have made up more than half of cases in recent reports [8, 85].

The case of Ghana illustrates well the gaps in our understanding of the ecology of CL in West Africa. Ghana was not considered endemic for CL until a first outbreak was reported in 1999, leading to an active screening in 2003. Thousands of suspect cases were reported in the Volta region, along the border with Togo [8], in a municipality including forest mountainous and savannah areas. However, very few cases were parasitologically confirmed and no species was identified until 2006, when a first isolation was made with an identification of *L. major* by sequencing the ribosomal RNA internal transcribed spacer 1 (*ITS1*) gene [86]. A second study was then performed in the same area but failed to detect any known *Leishmania* species [87]. Then, in 2015, Kwakye-Nuako et al. analysed three culture isolates from new human cases of CL. By sequencing ribosomal RNA internal transcribed spacer 1, ribosomal protein L23a intergenic spacer and RNA polymerase II large subunit (RNAPol II), they identified a new *Leishmania* strain with no match to any known species, clustering in the *Leishmania enriettii* complex [85]. A new study in 2021 also reported hundreds of cases in the Oti region, formerly part of the northern Volta region. PCR using the small subunit rRNA target enabled the confirmation of CL but not the identification of the responsible species [88]. In 2016, the new *Leishmania* subgenus *Mundinia* was created to harbour the *L. enriettii* complex, including the Ghana strain [89]. In 2023, one of the strains from the Volta region was sequenced and defined as the new *Mundinia* species, *Leishmania chancei* [90]. Overall, CL in Ghana seems very hard to confirm with current diagnostic tools, including molecular assays. Parasitological examination usually shows few amastigotes, and PCR is often negative, even on samples from patients with positive histopathology [8].

The majority of sand flies captured in Ghana belong to the genus *Sergentomyia*, whose ability to transmit CL to humans is still debated. However, small amounts of more common vectors like *Phlebotomus rodhaini* and *P. duboscqi* have also been captured [8, 85]. In the Volta region, sand fly captures performed in villages with incident human cases mostly yielded *Sergentomyia simillima*, *Sergentomyia ingrami* and *Sergentomyia africana* [8]. Blood meal analysis from these three species suggested that they fed on humans, chickens and goats [8]. In 2014, Leishmania DNA was detected by ITS1 PCR restriction fragment length polymorphism (RFLP) and sequencing in sand flies from the endemic Volta region, with *L. major* in *S. ingrami* pools, and *L. tropica* in one *S. ingrami* pool and in one *Sergentomyia hamoni* pool [91]. The *L. major* sequence was very close to the one isolated in a human sample by Fryauff et al. [86]. A new study in 2023 confirmed the presence of *L. major* in *S. ingrami* which had recently fed on humans. However, the most important source of meals for *S. simillima*, *S. africana* and *S. ingrami* was chickens [92]. DNA of *Leishmania* sp. was isolated in pools of *S. africana* in 2016 [93]. New species of *Sergentomyia* were found positive for *Leishmania* sp. in 2017 during catches in the South Dayi district of the Volta region, with *Sergentomyia similima*, *S. ghesquierei*, *S. antennata*, and *S. dureni* as well as *P. rodhaini* (33.33%) [94].

The inclusion of Ghana strains in the *Leishmania* subgenus *Mundinia* supports the hypothesis that CL in Ghana could be transmitted by midges, as these vectors are known to be associated with *Mundinia* [95–97]. Since then, several studies have investigated the potential role of *Culicoides* as vectors in Ghana. Catches in the Volta region have retrieved numerous populations of *Culicoides*, mostly *Culicoides grahami*, proposed as a potential vector [98]. Evidence of mammalian blood meals was shown in these *Culicoides* [99]. Further congress reports showed the ability of the human CL strain to develop in *Culicoides sonorensis* [100] and to be transmitted to mice [101].

Though *A. niloticus* and *M. natalensis* are present in the endemic communities of the Ho region, host competence tests did not show any survival of the Ghanaian *Leishmania* strains in these rodents [102].

### Central Africa

#### Cameroon

In Cameroon, CL is mostly observed in the northern savannah region of the Mokolo focus. Ulcero-crusty lesions seem to be the most frequent form [103].

The only identified species in human CL-typical lesions is *L. major* [103, 104], with the MON-76 zymodeme [105]. However, though *P. duboscqi* has been regularly captured near foci, there is no report of sand flies infected with *L. major* in this country. *Leishmania donovani* has been isolated in populations of *P. duboscqi* [106]. In 2017, an outbreak of Kaposi sarcoma (KS) was reported in children of the Mokolo district, with positive *L. donovani* PCR on skin samples from KS-like presentations including violaceous papulo-nodules and/or plaques, lymphoedema of the legs and lymphadenopathy [107]. Though histopathology was not performed, the clinical diagnosis of endemic KS was upheld, and the presence of *L. donovani* was therefore deemed a contamination. Therefore, the role of *L. donovani* in genuine human CL in Cameroon remains unclear. Large amounts of *Sergentomyia* have also been captured near human cases but without any reported *Leishmania* infection [108, 109].

There are currently no data on potential reservoirs in Cameroon, though rodents should be expected due to the presence of *L. major*. A study conducted in the southern forested area of Cameroon reported positive *L. major* PCR in gorilla faeces, along with microscopic visualization of both amastigote and promastigote forms [110]. However, as other authors have pointed out in comments, promastigotes should not be expected in mammalian hosts but only in sand flies, and amastigotes cannot be excreted in the faeces of mammals [111]. Bastien et al. [111] also discarded the hypothesis that sand flies ingested by gorillas would have led to the excretion of parasites in their faeces, as the digestion process would have destroyed them. In addition, this detection of *L. major* was reported in an area where it has never been reported in humans [110].

#### Chad

Although the presence of CL has been well demonstrated in Chad, there has been no species identification to date. *Phlebotomus duboscqi* and *S. schwetzi* have both been captured in important amounts in human dwellings in areas with clinical cases [82].

#### Southern Africa (Botswana, Namibia, Republic of South Africa and Zambia)

A clinical presentation of localized CL (LCL; oriental sore) is usually described in this region, with no cases of DCL or mucosal involvement [112].

CL has been reported in Namibia (then called South West Africa) since the 1970s. The *Leishmania* species involved in Namibia remains very hard to classify [113] and could belong to the *Leishmania* subgenus *Mundinia* [114]. Cases have also been reported in the north-west of the South African Cape Province [115]. Of note, there is one published report of a 15-year-old boy returning from South Africa with a CL ulcer and an identification of *L. major*, but the identification method was not detailed [116]. Lastly, a case of LCL caused by *L. tropica* was reported in a nurse returning from Botswana and Zambia [117].

*Phlebotomus rossi* has long been suspected as a vector in this part of SSA, while *Leishmania* amastigotes were detected by microscopy observation in nose tip imprints of local hyraxes (*Procavia capensis*), similarly to the Ethiopian setting. However, Grove et al. reported in 1989 that strains isolated in humans and *P. rossi* were different from those isolated in hyraxes [113].

CL in Austral Africa seems to occur in ecosystems of cliffs and rocky soils similar to those of the Ethiopian Highlands [115]. The eradication of jackals by humans has been linked to the proliferation of hyraxes and the subsequent emergence of CL in the dry cliff areas of Namibia and the north-west of the Cape Province [113].

#### Treatment

Concerning therapeutic options according to the different *Leishmania* species, most available data originate from East Africa. In Ethiopia, treatments for LCL caused by *L. aethiopica* include topical administration of paromomycin, cryotherapy, thermotherapy and intralesional or systemic administration of meglumine antimoniate [118]. Intravenous pentamidine and deoxycholate [119] or liposomal [27] amphotericin B are also known to be effective but are rarely used [27]. The evolution towards LCL, ML or DCL seems to be determined by both parasite and host factors [120].

Interestingly, in Kenya, high-dose intravenous sodium stibogluconate (18–20 mg/kg twice daily for 20 days) has been shown to be effective against cases of single-lesion *L. aethiopica* [121], but several relapses have been reported with *L. tropica* [28]. This species has been particularly associated with leishmaniasis recidivans, i.e. relapsing presentations after treatment with sodium stibogluconate [122]. Heat therapy has also been tried in this country but seems to be associated with clinical relapse [121]. However, the most common therapeutic option in Kenya remains intralesional injections of antimonials [32]. In Sudan, cases of both CL and ML are successfully treated with meglumine antimoniate (mostly with intravenous administration) or even orally administered ketoconazole [47, 50, 51].

In West Africa, meglumine antimoniate (usually intralesional) is the most frequent option against *L. major*, as indicated in reports from Senegal [55, 56], Mauritania [77] and Niger, where other options, such as oral ketoconazole and oral metronidazole, are also used [81, 123]. Concerning the recently described species involved in Ghana, most patients seem to use traditional remedies such as plants or homemade thermotherapy with hot water or stones [88]. In Cameroon, where the only isolated species is *L. major*, topical amphotericin B deoxycholate and oral metronidazole have been used with success [103].

In Southern Africa, very few data were available, with some reported cases of surgical treatment (usually not recommended in CL) [113], topical paromomycin and oral fluconazole [116].

#### Diagnosis

The identification of *Leishmania* species relied on various techniques. Earlier studies were mostly based on detection of specific isoenzymes and zymodeme determination. Since the 2000s, the use of PCR has become more frequent, followed by either RFLP or sequencing. When performed, PCR in humans used several types of samples, including skin [70, 124, 125] or mucosal [50] biopsies, skin scrapings or smears [126], ulcer aspirates [127] and, more recently, tape discs or filter papers [48, 88]. The ITS gene was the most frequently used target.

A detailed table of diagnostic methods used in original reports included in this study is available as Supplementary file S15.

## Discussion

This review highlighted heterogeneous results in terms of available data for each of the transmission cycle components. Many entomological works were retrieved but many fewer studies on reservoirs. Clinical and therapeutic data were collected through case series or descriptive reports, but we could not retrieve any clinical trial comparing treatments for sub-Saharan *Leishmania* species or any large systematic study comparing the clinical features for each species. An upward trend was observed in the number of publications after 2010, indicating possible advances in this field of research. However, we could not retrieve any report for several countries which are known to be endemic (i.e. Guinea, Ivory Coast or the Central African Republic) [3], suggesting that large areas remain to be mapped for *Leishmania*, vector and reservoir species.

Our review included many more studies from East Africa than from West Africa. CL and VL benefit from important research efforts in Ethiopia, Kenya and Sudan. Several factors can explain this difference, such as the high prevalence of VL in East Africa, as well as higher development indicators in Ethiopia and Kenya than in most countries of West Africa [128] and a long tradition of research in leishmaniasis, inherited from British colonial medicine. This region is a good example of how the understanding of *Leishmania* species and their respective parasitic cycles can shape local human epidemiology. Indeed, *L. aethiopica*, *L. tropica* and *L. major* all present different vectors and reservoirs and are present in different environments. *Leishmania tropica* and *L. aethiopica* were not distinguished from each other until the 1970s [20]. It has been suggested that ancestral *Leishmania* developed into *L. tropica* and *L. aethiopica* through their presence in the lowland or the highland environments of East Africa [16]. From a clinical perspective, setting these two species apart is justified by the clinical challenge of treating *L. tropica*. CL caused by *L. donovani* could be underestimated in East Africa. Only a few cases have been reported in Kenya, though CL and VL areas overlap in this country. Hybrids between *L. donovani* and other species (*L. tropica* or *L. major*), such as those observed in Sudan, may lead to the emergence of *L. donovani* CL in other areas of East Africa.

CL and VL are caused by different *Leishmania* species, corresponding to different vectors and reservoirs. However, the endemic areas of these two diseases can overlap. The presence of hybrids between *Leishmania* species with either cutaneous or visceral tropism has been demonstrated in Sudan and Ethiopia [129]. Although out of the scope of this study, such hybrids have also been reported in Sri Lanka [130, 131]. Therefore, the distinction between these two clinical entities might not be as clear as previously thought, and would have important implications in terms of human epidemiology and case management. Hybrids from *L. aethiopica*, *L. tropica* or *L. major* and *L. donovani* could gain adaptation characteristics to different vectors and could therefore spread in areas where the original species was not found. It has also been suggested that interactions between *Sauroleishmania* such as *Leishmania tarentolae* and pathogenic species such as *L. donovani* could lead to new hybrids [132].

This review shows how Sudanese ML might be another example of a fluid border between CL and VL. Indeed, several authors have suggested that Sudanese ML occurs in patients with previous history of VL. This hypothesis is supported by the identification of *L. donovani* in mucosal lesions and by patients’ history. However, it is contrary to what is observed in the Americas, where ML is usually preceded by CL [133]. Moreover, *L. major* has also been identified in mucosal lesions in Sudan, suggesting that both CL and VL could precede mucosal involvement. Variations in immune responses based on host factors could explain discrepancies in clinical evolution after an initial episode of CL or VL. Unfortunately, most of the studies discussing the continuum between CL, VL and ML in Sudan are ancient and did not explore these aspects.

This study allowed us to identify several research gaps in West Africa. With the notable exception of Ghana, CL is mostly reported in the semi-Sahelian dry areas of this subregion. The parasitic cycle of *L. major* has been well described in Senegal, with the involvement of *P. duboscqi* as vector and rodents as reservoirs. However, assumptions are made that similar ecologies in neighbouring countries will lead to a similar cycle. Although *L. major* has also been identified in Mali, Niger and Burkina Faso, PCR is rarely performed in these countries, and a systematic screening could identify unsuspected *Leishmania* species, particularly in more humid and forested areas at the fringe of the Sahelian zone. Vector and reservoir species involved in the transmission to humans should be confirmed in countries where few veterinary or entomological studies have been conducted, such as Chad, Mauritania, Niger or Gambia. Although *L. major* has been reported in Mauritania, *L. infantum* was also described in one study. This species can cause both VL and CL in the Mediterranean [134], but was only reported in cases of CL in Mauritania. This finding is unique in this area and deserves confirmation, as it would widen the list of potential vector and reservoir species in this country.

Ghana represents one of the most complex settings in terms of the *Leishmania* parasitic cycle. The identification of the *Leishmania* species responsible for human lesions in Ghana remains challenging. *Leishmania major* [86] was once implicated, while more recent studies suggested the involvement of a rare strain belonging to the *Leishmania* subgenus *Mundinia* [85, 99]. Several species might be involved. Another hypothesis is that previous identification of *L. major* lacked precision and was unable to detect a new, undescribed species. *Leishmania major* is usually reported in dry areas but has also been detected in the forest environment of the Ghanaian Volta [91]. The speciation of *L. enriettii* remains doubtful, and this species is usually not pathogenic for humans. The *Leishmania* subgenus *Mundinia* as a whole has been created to regroup incompletely understood *Leishmania* strains which are supposed to belong to an archaic group of proto-*Leishmania* parasites [114]. It is possible that a new entity, an “African forest leishmaniasis”, might exist in the forested areas of Ghana, and should be looked for in similar environments of neighbouring countries, such as in Togo. This specific kind of CL could involve vectors and reservoirs that are very different from those of Sahelian or East African CL. Further studies are needed to confirm the role of midges as potential vectors and chickens or rodents as potential reservoirs. The transmission dynamics in this Ghanaian focus are also unclear, as detection rates seem to be inconsistent. It is not known whether this focus was active but undetected before the 2000s or whether CL appeared in a new area with a previously non-immune population, perhaps after ecological changes.

In Burkina Faso, the possible role of dogs as reservoirs of CL has been suggested by studies conducted in the Bobo-Dioulasso area [73]. These findings are not in line with the usual assumption that rodents are the only reservoir for CL in West Africa and that dogs only serve as reservoirs for VL. Because of their proximity to humans and their presence in urban areas, the role of dogs should be further investigated in SSA, as it would mean a major shift in the risk of occurrence of human CL in towns. To date, CL remains mostly a rural disease in SSA. However, ecological disruptions as well as uncontrolled urbanization due to massive population displacement could result in changes in sand fly feeding behaviour and favour the emergence of CL in new settings with new mammal species as reservoirs. For example, hares (*Lepus granatensis*) infected with *L. infantum* were implicated as potential new reservoirs after a VL outbreak in the Madrid region in Spain [135]. This role of dogs should be investigated not only in Burkina Faso but in the whole West Africa subregion–*L. major* ecological area. Concerning vectors, *P. duboscqi* is still seen as the vector responsible for the transmission of *L. major* in this area. *Sergentomyia* sand flies such as *S. schwetzi* have also been suspected, due to significant captures close to human CL cases and due to their anthropophilic behaviour. However, experimental tests have shown that *S. schwetzi* is not a competent vector for species such as *L. major*, discarding its possible role in this parasitic cycle in West Africa [136]. Overall, West Africa should benefit from much more significant research efforts on CL, as this disease is probably under-diagnosed in this region [3] and its transmission dynamics are still incompletely understood.

Environmental and human factors can lead to the emergence of new CL foci. In Sudan, a large outbreak occurred near Khartoum which affected patients of all ages, suggesting the introduction of a new disease in a previously non-immune population [47]. In Ethiopia, global warming could induce a decrease in rainfall which would lead to the emergence of *L. aethiopica* in the lowlands [17]. The impact of these climatic changes should be studied in other areas, for example in West Africa, where increased dryness could make some areas more suitable for the *P. duboscqi*–*L. major* complex. Elimination of rodents, which are important reservoirs in both West and East Africa, could also shift the transmission towards other hosts and allow CL to emerge in more anthropized environments. Overall, very few studies have investigated the role of climatic factors in the epidemiology of CL in SSA, compared to the many works performed in South America [137], North Africa [138] or Asia [139]. It is noteworthy that many other factors, including political migrations [140], malnutrition [141], sleeping behaviours [142], agricultural practices [142] or deforestation [143], can play a part in CL emergence.

From a clinical point of view, this study highlights the presence of DCL in many sub-Saharan countries. DCL is a very peculiar entity in which the immune response is not able to induce an effective granulomatous control, leading to the constitution of multiple anergic nodular lesions [5]. The treatment of DCL is often very difficult, due to the ineffective host response and the high parasitic load. Some authors tend to mistake it for disseminated CL (multiple lesions with low parasitic load, due to specific *Leishmania* strains but with no defect in immune response) [6], especially when histopathology is not available. We found reports of DCL in two main foci, one in East Africa with Ethiopia and Sudan, the other one in West Africa with Senegal and Burkina Faso. *Leishmania aethiopica* was incriminated in the Ethiopia focus while *L. major* was identified in both foci. As DCL can be mistaken for lepromatous leprosy, it is important for specialists to know of its existence in their area of practice. We found several publications reporting original presentations in East Africa (mycetoma-like, sporotrichoid, etc.). We found no such description in West Africa, but this might be due to an under-reporting bias in this region with fewer publications.

The diagnostic confirmation of CL and the identification of specific *Leishmania* species is one of the main barriers highlighted in this review. The identification of *Leishmania* species, whether in human or animal samples, has long relied on isoenzyme analysis, which is cumbersome and does not allow for precise identification. Only recently has PCR allowed for easy and precise analysis of the different strains involved. However, molecular biology remains unavailable in many settings in SSA, which makes the cartography of *Leishmania* species hard to complete. The use of PCR followed by sequencing and phylogeny is also necessary to differentiate between strains within the same species. Discrepancies can be observed between ancient findings established with isoenzyme analysis and recent ones determined with PCR. For example, in a mucosal biopsy in Sudan, Ibrahim et al. [144] reported a zymodeme that was unrelated to any strain isolated in the region [144]. On the other hand, PCR of the kinetoplastic deoxyribonucleic acid (kDNA) gene identified *L. donovani*. The quality of skin sampling is also of paramount importance. For example, one of the first studies on the undetermined species in Ghana was based on formalin-fixed biopsies, which implied a possible denaturation of parasitic DNA [86]. Simple and non-invasive sampling techniques such as filter paper or tape discs can be used in humans, even in remote settings. Although the use of cotton swabs for CL PCR is common in South America, we found no such report in our review. Swabs could provide an easy sampling method for CL ulcers in SSA, followed by PCR and sequencing [145].

However, even when PCR is used, discrepancies can be observed with different target genes (for example, ITS or RNA Pol II) and methods for species identification, such as RFLP or Sanger sequencing. For example, strains of CL from Ghana have successively been identified as *L. major* by sequencing of the ITS gene [86], then as a strain clustering in the *L. enriettii* complex by using RFLP of the *ITS1*, ribosomal protein L23a (*RPL23a*) and RNA Pol II genes [85], then as an unidentified strain when using species-specific nested PCR of the 16S rRNA gene [88], and then as *L. chancei* by sequencing of the *RPL23a* and RNA Pol II genes [90].

Concerning treatments, meglumine antimoniate was by far the most frequently used drug. Its intralesional route is often preferred due to reduced costs and lower toxicity. Efficacy was reported against *L. aethiopica* and *L. major* in East Africa, and against *L. major* in West Africa. Other options were also reported, such as oral ketoconazole against *L. major* in Sudan and in Niger, or oral metronidazole against *L. major* in Cameroon and in Niger. Meglumine antimoniate or sodium stibogluconate were described as less effective against *L. tropica*. Other options such as cryotherapy or heat therapy were also associated with relapses. Other drugs such as pentamidine [20] or liposomal amphotericin B could be investigated [27] against *L. tropica*, though their availability (pentamidine) and cost (liposomal amphotericin B) may prevent their use in some areas. Although guidelines for the management of CL do not recommend waiting for the determination of species before treating a patient [146], differences in drug sensitivity justify the mapping of species across different areas in order to guide therapeutic options in the field. Data concerning therapeutic options were often very limited, highlighting the need for further interventional studies on the management of CL in SSA.

This work has several weaknesses. Some studies could have been missed by the queries. Veterinary sources could have been forgotten in the inclusion process. This study was focused on the implications of data on human health, and purely veterinary or ecological data could have been omitted. However, this study is the first to summarize data on *Leishmania*, vectors and mammal reservoir species involved in CL in SSA. The inclusion of grey literature is an important strength due to the presence of a significant amount of relevant but unpublished data.

## Conclusions

In conclusion, this study highlights the importance of filling some research gaps regarding the species of *Leishmania*, vectors and mammal reservoirs involved in the transmission of CL in SSA. The implications of *L. major* should be confirmed in semi-Sahelian areas of West Africa, while the question of African forest CL should benefit from more investigations. The role of hybrids between different species should be investigated in East Africa, along with the natural history of mucosal involvement. Reservoirs of *L. tropica* in East Africa also remain mostly undetermined and deserve further studies. The impact of ecological and climatic changes on the transmission dynamics of CL in SSA should be studied through intersectional collaborations between climate and One Health specialists. The recent development of molecular biology capacities in SSA fostered by the COVID-19 pandemic should improve the diagnostic capacity of CL and, through the identification of the *Leishmania* species involved, will help to guide the therapeutic strategy for incident cases of CL in specific areas.

### Supplementary Information

Additional file 1.

Additional file 2.

Additional file 3.

Additional file 4.

Additional file 5.

Additional file 6.

Additional file 7.

Additional file 8.

Additional file 9.

Additional file 10.

Additional file 11.

Additional file 12.

Additional file 13.

Additional file 14.

Additional file 15.

## Data Availability

The datasets generated and/or analysed during the current study are available from the corresponding author on reasonable request.

## Abbreviations

Ab-ELISA: Antibody enzyme-linked immunosorbent assay
CL: Cutaneous leishmaniasis
DCL: Diffuse cutaneous leishmaniasis
HIV: Human immunodeficiency virus
ITS: Internal transcribed spacer
kDNA: Kinetoplastic deoxyribonucleic acid
KS: Kaposi sarcoma
LCL: Localized cutaneous leishmaniasis
ML: Mucosal leishmaniasis
PCR: Polymerase chain reaction
PKDL: Post Kala-Azar dermal leishmaniasis
RFLP: Restriction fragment length polymorphism
RNA POL: Ribonucleic acid polymerase
RPL23A: Ribosomal protein L23a
SSA: Sub-Saharan Africa
VL: Visceral leishmaniasis
